# Distilling vector space model scores for the assessment of constructed responses with bifactor Inbuilt Rubric method and latent variables

**DOI:** 10.3758/s13428-021-01764-6

**Published:** 2022-01-11

**Authors:** José Ángel Martínez-Huertas, Ricardo Olmos, Guillermo Jorge-Botana, José A. León

**Affiliations:** 1 Faculty of Psychology, Universidad Autónoma de Madrid, Calle Iván Pavlov, 6, Ciudad Universitaria de Cantoblanco, 28049 Madrid, Spain; 2 Faculty of Human and Social Sciences, Universidad Pontificia de Comillas, Madrid, Spain; 3 Faculty of Psychology, Universidad Complutense de Madrid, Madrid, Spain

**Keywords:** Inbuilt Rubric, Vector space models, Bifactor, Measurement models, Validity, Constructed responses

## Abstract

In this paper, we highlight the importance of distilling the computational assessments of constructed responses to validate the indicators/proxies of constructs/trins using an empirical illustration in automated summary evaluation. We present the validation of the Inbuilt Rubric (IR) method that maps rubrics into vector spaces for concepts’ assessment. Specifically, we improved and validated its scores’ performance using latent variables, a common approach in psychometrics. We also validated a new hierarchical vector space, namely a bifactor IR. 205 Spanish undergraduate students produced 615 summaries of three different texts that were evaluated by human raters and different versions of the IR method using latent semantic analysis (LSA). The computational scores were validated using multiple linear regressions and different latent variable models like CFAs or SEMs. Convergent and discriminant validity was found for the IR scores using human rater scores as validity criteria. While this study was conducted in the Spanish language, the proposed scheme is language-independent and applicable to any language. We highlight four main conclusions: (1) Accurate performance can be observed in topic-detection tasks without hundreds/thousands of pre-scored samples required in supervised models. (2) Convergent/discriminant validity can be improved using measurement models for computational scores as they adjust for measurement errors. (3) Nouns embedded in fragments of instructional text can be an affordable alternative to use the IR method. (4) Hierarchical models, like the bifactor IR, can increase the validity of computational assessments evaluating general and specific knowledge in vector space models. R code is provided to apply the classic and bifactor IR method.

## Introduction^1^

Computational semantic measures are relevant to obtain indicators of different psychological constructs (e.g., Kjell et al., 2019). The general purpose of these methods is to detect indicators in the utterances of the people being assessed. This is especially valuable in academic assessment (e.g., Bejar et al., 2016; Landauer et al., 2007; McNamara, 2007; Shermis & Burstein, 2013; Yan et al., 2020). The automation of this assessment has caused a significant shift from traditional or classical approaches to psychological assessment using written materials. An efficient automatic system should identify some relevant *constructs* or *trins* (the object of the assessment) from some *indicators* or *proxies* (observable features in text). This consideration is analogous to the psychometric process of creating a psychological task or test where one should evaluate (a) whether the constructs can be a relevant instance of the object of assessment, and (b) whether the indicators are appropriate to infer such constructs. These considerations are in line with the need to gain reliability and validity in the computational assessment of texts (e.g., Attali, 2014; Bejar et al., 2016; Koskey & Shermis, 2014; Rupp, 2018).

While several possible indicators can be retrieved from a text (including pattern detection, syntactic and logical sequences, etc.), we are going to focus on the semantic cues that arise from vector space models. In any case, these semantic cues can also be merged within a larger model taking advantage of several indicators. As is known, vector space models allow us to represent words and texts in a multidimensional vector space that maps the knowledge of a specific linguistic corpus (see Günther et al., 2019; Jones et al., 2018; or Jorge-Botana et al., 2020, for a recent review on vector space models). In the evaluation of constructed responses, as in automated summary evaluation, text responses are represented in the vector space. Here, the semantic dimensions of those vectors are the indicators of the text responses. In vector space models, the evaluation of constructed responses is usually made by comparing the latent vector that represents the text response to be assessed with the latent vectors of “ideal” responses or parts of those ideal responses^2^. It is important to highlight that these vectors are latent in the sense that their coordinates (dimensions) have no meaning themselves (that is, vectors are just comparable but not interpretable). Nonetheless, some proposals have been made to transform the latent nature of these vector spaces into semantic spaces whose coordinates could have a priori explicit semantic meanings, such as the meaning of the important concepts we want to identify and evaluate in texts (e.g., Hu et al., 2007). One of these proposals is the Inbuilt Rubric method, named for its capacity to transform some coordinates of the original vector space into a rubric to evaluate semantic concepts. This method endows the dimensions of the vector space with semantic meanings determined a priori by the designer of the rubric*.* This method further makes vectors more than a meaningless set of coordinates as it generates comparable and interpretable coordinates in the vector space (Jorge-Botana et al., 2019; Martínez-Huertas et al., 2018, 2019, 2021; Olmos et al., 2014, 2016).

Based on the previous theoretical background, the present study aims to make a formal proposal about the combination of computational scores and standard psychometrics^3^. In this respect, this study illustrates how standard psychometric procedures can validate and even improve the performance of computational methods like Inbuilt Rubric using a latent variable framework. Specifically, we will combine the strengths of semantic measures from vector space models (and some algebraic manipulation of them) and their underlying measurement models to validate computational psychoeducational assessments. In sum, this study defends the necessity of using a validity-centered approach to gather evidence in favor of computational scores to measure constructs from written materials (see a similar rationale in Attali, 2014; Bejar et al., 2016; Koskey & Shermis, 2014; Rupp, 2018). Classic psychometric tools like latent variable models (e.g., structural equation models [SEMs]) can be used to isolate and validate the constructs suggested by the rubric designers and the scores of the Inbuilt Rubric method. This paper is organized as follows: first, we provide a brief introduction to vector space models and psychoeducational assessment; second, we present the fundamentals of the assessment by means of Inbuilt Rubric in some of its configurations; and third, the combination of latent models with Inbuilt Rubric is proposed and empirically tested in a study on automated summary evaluation.

### Automated summary evaluation for psychoeducational assessments

Different studies have shown the relevance of summarizing in evaluating comprehension and text-based learning (e.g., Franzke et al., 2005; Hong, 2016; León et al., 2006; Saddler et al., 2017; Stevens et al., 2019; Sung et al., 2016; Wade-Stein & Kintsch, 2004). Summarizing requires the capacity to generalize, synthesize, and write coherently, which implies profound comprehension, incorporating previous knowledge and active processes such as inference-making (van Dijk & Kintsch, 1983). This theoretical model assumes that summarizing is essential for comprehension since it supposes the extraction and elaboration of text content to generate rich representations of concepts. In this vein, some authors have argued that multiple-choice tests based on recognition memory cause less deep learning than constructed responses based on memory recall (e.g., Millis et al., 2007; Shapiro & McNamara, 2000). However, the evaluation of constructed responses such as student summaries requires much effort and time recourses for the evaluators. That is why developing automated assessments of computational models of language is so important. Summary Street, WriteToLearn, and G-Rubric are some examples of applications employing latent semantic analysis (LSA) in psychoeducational assessment. They all teach how to make a summary from expository texts providing individualized feedback to students (Foltz et al., 2013; Kintsch et al., 2007; Olmos et al., 2016). Many other applications of automated summary evaluation can be found in the literature (e.g., Crossley et al., 2019; Dascalu et al., 2015; Li et al., 2018; Li & Graesser, 2020; Mintz et al., 2014; Ruseti et al., 2018).

In Foltz et al. (2013), there is an extensive description of the indicators of the student summaries to be evaluated. Among others, the main constructs (indicators appear within parentheses) are grammar (grammatical errors, error types, etc.), style (topic development, organization, etc.), mechanism (punctuation, spelling, capitalization, etc.), lexical sophistication (word variety, technical words, etc.), and content (presence of topics). As stated previously, this study is focused on content, where vector space models have been preeminently used. One of the most popular vector space models to determine the content of texts, especially for the assessment of constructed responses, is LSA (Deerwester et al., 1990; Landauer et al., 2007; Landauer & Dumais, 1997). LSA extracts and represents the meaning of words in a multidimensional space. The semantic representations are obtained after applying dimensionality-reduction algebraic methods (like singular value decomposition—SVD) to large corpora to represent the meaning of words in a reduced number of dimensions (usually in a 300-dimensional space). The LSA model has been studied extensively for more than 20 years in a variety of tasks, and it shows great ability to emulate semantic human behavior (involving semantic judgments, classification tasks, search engines, relevant elements in texts, etc.). See Landauer and Dumais (1997) or Landauer et al. (2007) for a complete description of LSA.

A key aspect of vector space models like LSA is that one can represent texts as vectors by means of a simple projection in the semantic space. Thus, similarities among vector representations are computed with distance metrics. In psychoeducational assessments, distances between the vectors of student summaries and gold summaries (ideal summaries written by experts) are computed. Golden summaries can be different depending on the method used, as they can be whole gold summaries (ideal summaries to compare with) or partial summaries (paragraphs or sentences extracted from whole gold summaries describing different topics; Dessus & Lemaire, 1999; Franzke et al., 2005; Kintsch et al., 2007; Magliano & Graesser, 2012; Martínez-Huertas et al., 2021), or a set of gold summaries pre-rated with scores from different expert (Olmos et al., 2009b). In the field of automated summary evaluation, León et al. (2006) analyzed six different LSA methods. These methods were holistic, such as the cosine between student summaries and instructional texts, or componential, such as the mean cosine between each sentence from student summaries and some representative sentences from instructional texts. León et al. (2006) found that holistic methods were more accurate than componential methods. Later, Olmos et al. (2009b) extended those results by comparing more complex holistic LSA methods such as best-dimension reduction that computes the cosine (a measure of distance) using only the relevant LSA dimensions to evaluate summaries or the Euclidean distance that combines cosine and vector length measures. Olmos et al. (2009b) discovered that the performance of LSA could be increased using just the relevant dimensions of the latent semantic space like the best-dimension reduction method (Hu et al., 2007). These results showed that LSA methods were accurate for measuring the overall quality of student summaries, especially when the LSA’s semantic space was honed. Similarly, other studies refined the parameters of the latent semantic space for automated summary evaluation (e.g., Jorge-Botana et al., 2010; Olmos et al., 2009a), and its applicability has been widely tested (e.g., Li et al., 2016b, b; Li et al., 2018; Malladi et al., 2010; Olmos et al., 2011, 2013).

Nevertheless, this previous research was all conducted using latent semantic spaces. Inspired by the proposal by Hu et al. (2007), Olmos et al. (2014) proposed the Inbuilt Rubric method, which transforms the latent semantic space into a meaningful one. As we will see in the next section, its logic is based on the mapping of an assessment rubric’s items into vector space dimensions. Thus, the simple projection of constructed responses can provide information about the presence and absence of content without comparing vectors with latent meanings^4^.

### Mapping assessment rubrics into vector spaces using the Inbuilt Rubric method

Inbuilt Rubric is a recently developed LSA method that converts the latent meaning of some dimensions of LSA’s vector space into the meaning of an academic assessment rubric’s items (Olmos et al., 2014, 2016). The main advantage of this method is that the meaning of a text is estimated with a simple projection in that new meaningful vector space. The resulting vector of the projection of a constructed response has information about the presence or absence of the items of the rubric. The scores or coordinates of each dimension show the extent to which a text covers the knowledge domain pertaining to each item. Inbuilt Rubric method can be considered a model that maps assessment rubrics into vector space models (a more specific explanation of this method can be found in Olmos et al., 2014, 2016, or in Martínez-Huertas et al., 2021). Previous research has shown that the overall scores of the Inbuilt Rubric method demonstrate better performance than the Golden Summary method using the same original LSA semantic space (Martínez-Huertas et al., 2018, 2019; Olmos et al., 2016). Similar results have been observed for the specific concept scores of Inbuilt Rubric compared to the partial content similarity method (Martínez-Huertas et al., 2021). A brief description of this method shall now be provided.

In the psychoeducational assessment of constructed responses, the Inbuilt Rubric method requires different sequential steps: (1) A “rubric” is established to define the target concepts whose relevance in the student texts is to be scored (for an example, see the *Instruments* section). Although this is done to evaluate constructed responses, we could select other target concepts to study other types of stimuli. (2) A semantic space of LSA is generated using standard procedures. (3) Different lexical descriptors are chosen by consensus to make a representation of each target concept of the “rubric” (e.g., for the target concept “Darwin’s expedition,” lexical descriptors such as “Beagle” or “Galapagos Islands” are good candidates to adequately represent the concept). Therefore, these lexical descriptors must be words represented in the LSA space that are brought together to form each target concept. Each word has its own vector in the LSA space, so adding them together results in the vector that represents the concept. These vectors of target concepts are collected in the first columns in a matrix called ***β***. To complete the ***β*** matrix up to the number of dimensions of the original space (usually 300), it is randomly filled with column vectors from the standard basis. As a result, this matrix contains the basis of the new meaningful semantic space (wherein the first columns are meaningful); and (4) To change the space to have the new meaningful basis, a matrix computation of the ***β*** matrix is performed. Such a matrix computation involves just a matrix rotation where the ***US*** matrix of the original vector space whose first *k* dimensions pretend to evaluate the target concepts. This operation transforms the original “latent” semantic space into a new “meaningful” one.

Let us examine this procedure in detail using an example from the present study. As previously stated, the Inbuilt Rubric method requires the generation of matrix ***β*** that represents *k* target concepts or items of the designed assessment rubric (one item corresponds to one vector). These dimensions are computed as the sum of the vectors pertaining to each set of lexical descriptors of each concept (later, these vectors are normalized). For example, the dimension “Darwin’s theory” that will be used in the present study is computed as the sum of the vector depicting its set of lexical descriptors—“selection,” “natural,” and “evolution”—which would ideally represent such a concept. The evaluation of five concepts would require a matrix with five vectors that should be complemented with *p*-*k* vectors of the original latent semantic space to equal the number of dimensions, *p*, of the original vector space (this is done by adding vectors from the standard basis) (see Hu et al., 2007, p. 414). As previously stated, the generation of these *k* vectors of the matrix ***β*** requires the selection of some lexical descriptors to represent the target concepts from the instructional text. This selection calls for a systematic and exhaustive consensus between researchers to have a good definition of each concept in the initial latent semantic space. For example, if one wants to evaluate “the journey that Darwin made around the world,” the word “Beagle” may appropriately represent part of that concept. Therefore, it is a requirement to check what is understood by “Beagle” in the semantic space before including such a lexical descriptor in a vector that maps that concept. Thus, this process is not automatic and is, in some sense, arbitrary as it depends on the knowledge of the designer. This is why we are also proposing an alternative procedure in this study to automatically generate the vectors of matrix ***β***.

**Fig. 1:**
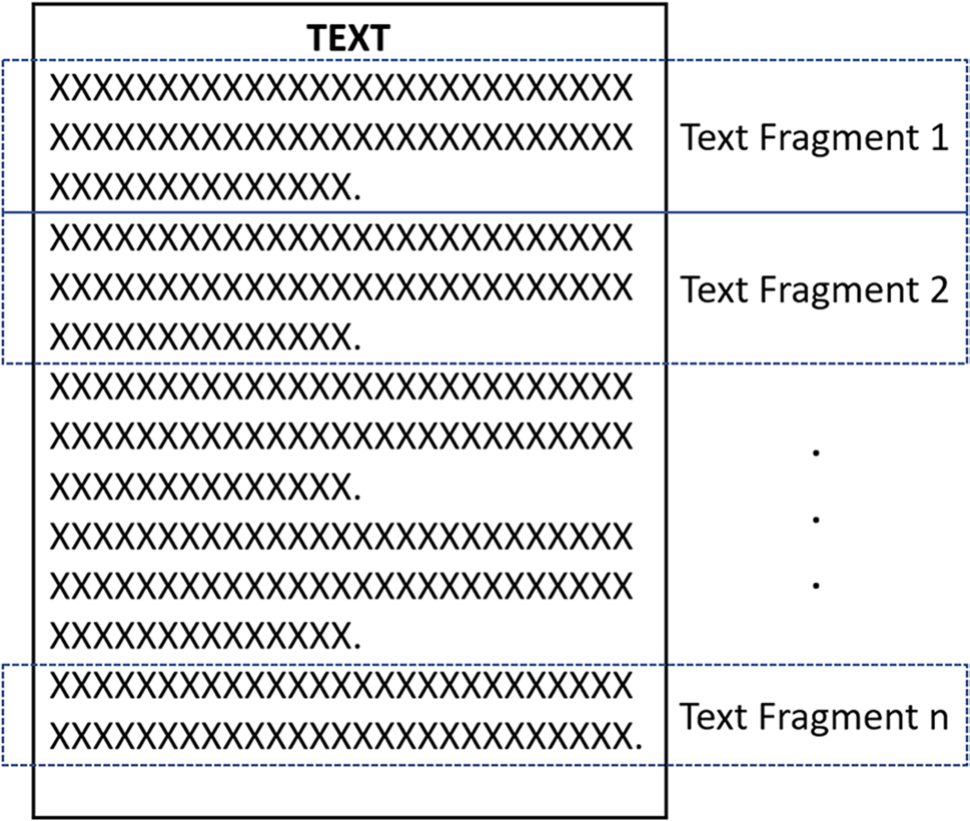
Example of text fragments of a hypothetical instructional text. *Note*: Instructional texts of this study had clear divisions for the different conceptual axes

Such a new version of the Inbuilt Rubric method uses nouns^5^ embedded in fragments of the instructional text in matrix ***β***. While the classic ***β*** matrix is generated through the selection of lexical descriptors, this alternative procedure avoids such selection and adopts a more automatic process. Figure 1 represents a hypothetical example of the extraction of such text fragments. The essence of this procedure is similar to other previous methods performing content-detection tasks such as partial content similarity or partial golden summaries (explained previously) that select relevant fragments of the original text stimuli (Dessus & Lemaire, 1999; Franzke et al., 2005; Kintsch et al., 2007; Magliano & Graesser, 2012; Martínez-Huertas et al., 2021), but it only uses nouns embedded in those fragments of the instructional text. In this version, a ***β*** matrix is created using different *k* vectors that are compounded by different number of nouns for each concept to be evaluated. This version is more automatic and does not require decision-making about the lexical descriptors to be used to transform the latent semantic space. Fundamentally, this version of the Inbuilt Rubric method only requires the selection of different text fragments that represent the target concepts to be evaluated. Such fragments can be part of the instructional text or other materials that are longer and more complex than the lexical descriptors.

As previously mentioned, the Inbuilt Rubric method follows a confirmatory strategy that imposes the conceptual structure of a rubric on the vector space, transforming some of its dimensions into concepts. To do this, Inbuilt Rubric uses a new basis, the aforementioned ***β*** matrix, with vectors that represent the concepts of the rubric. Matrix ***β*** is used to transform the latent vector space into a one in which some dimensions capture the meaning of some target concepts. While this transformation involves a simple rotation that makes simple projections possible (i.e., the original semantic distances remain), it is necessary to orthogonalize the vectors of matrix ***β*** before. Thus, the dimensions of matrix ***β*** (that is, the target concepts to be evaluated and the rest of the matrix ***β***) are artificially forced to have no common variance between them. This aspect has been considered one of the main advantages of the Inbuilt Rubric method as it avoids multicollinearity (Martínez-Huertas et al., 2021). However, recent proposals have tried to analyze the common variance that exists between the evaluated concepts in the Inbuilt Rubric method (Jorge-Botana et al., 2019). In the next section, we will introduce how it is possible to create a general factor of knowledge in the vector space, and we will also raise some questions about the meaning of such a general factor.

It is worth mentioning that the concepts of the vector space are established a priori by means of the Inbuilt Rubric method. Other proposals have used varimax rotations in the term loadings of the vector space to interpret the meaning of some dimensions of the LSA semantic space (Evangelopoulos, 2013; Evangelopoulos et al., 2012; Evangelopoulos & Visinescu, 2012; Kallens & Dale, 2018; Kundu et al., 2015; Visinescu & Evangelopoulos, 2014). While these authors also projected documents in rotated semantic spaces and could interpret the meaning of some dimensions, their strategy was more exploratory, and it is thus not possible to determine what concepts to evaluate. On the contrary, the Inbuilt Rubric method imposes concepts onto the vector space, and it can be used as a map for knowledge representations of different concepts established a priori.

### The lack of a general factor in vector space models: A measure of general knowledge?

In some sense, the concept dimensions used in the Inbuilt Rubric method can be considered orthogonal specific/group factors in a confirmatory bifactor model. As previously explained, the concept dimensions of the vector space are orthogonal, so they do not share common variance. Jorge-Botana et al. (2019) assumed that such common variance could be a valuable measure of general knowledge and studied how to manage the common variance that remains between the concepts of the rubric after Inbuilt Rubric manipulation. The authors introduced a complement into the classic Inbuilt Rubric in which an additional vector is estimated in matrix ***β*** that represents the common variance of the individual vectors of the rubric concepts. That new (general) vector is extracted through an exploratory factor analysis of the vector representations of the lexical descriptors. In this parametrization of the Inbuilt Rubric method, the lexical descriptors of each concept are split into *i* sets (here, *i* = 1, 2) and are represented in the semantic latent space. For example, if a rubric has four concepts, then eight vectors would be involved in this procedure (Jorge-Botana et al., 2019 also suggested other potentially useful methods for this purpose). An exploratory factor analysis is carried out, and the factor scores of the one-dimensional solution are estimated. Thus, the general factor is computed by weighting such vector representations by their respective factor loadings (*λ*_*ki*_):

1 $$General\ factor={\lambda}_{1i}\ast {Dim}_{1i}+{\lambda}_{2i}\ast {Dim}_{2i}+\dots +{\lambda}_{ki}\ast {Dim}_{ki}+ erro{r}_{ki}$$

where *k* is the number of concepts to be evaluated, and *i* is the number of the partitions of the lexical descriptors of each concept (in this parametrization: *i* = 1, 2). This general factor is included in the *k*+1th position of matrix ***β*** before the vectors of the rubric concepts, and it represents the common variance between the vectors of the rubric concepts. Finally, the vectors of matrix ***β*** are also orthogonalized.

**Fig. 2:**
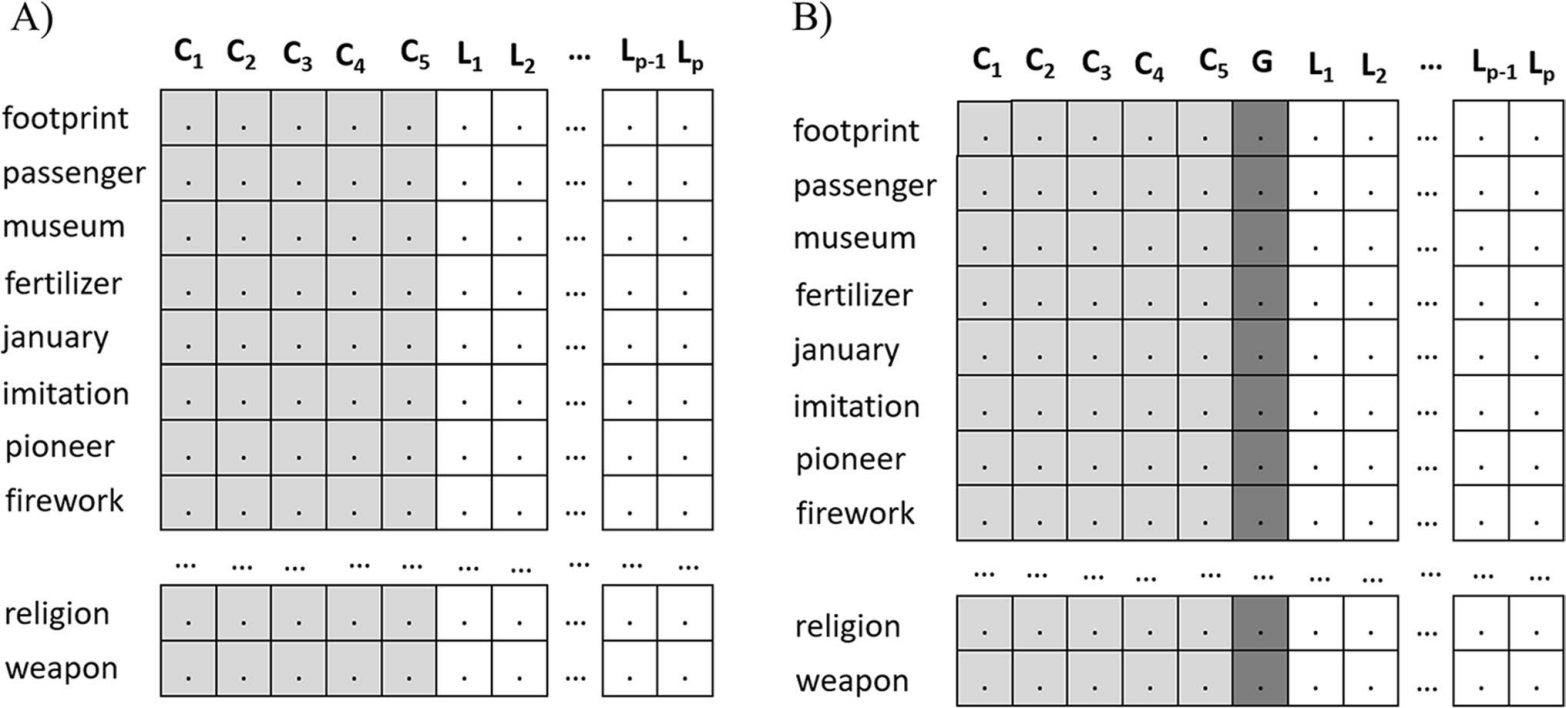
Graphical representations of three meaningful dimensions of **a** Inbuilt Rubric and **b** bifactor Inbuilt Rubric methods. *Note*. C_1_-C_5_ are the rubric concepts, G is the general factor, and L-L_p_ are latent dimensions of the original vector space. Dimensionality of the resulting vector spaces is *p* (usually, *p*=300). The number of interpretable dimensions (*k*) differs between the Inbuilt Rubric and the bifactor Inbuilt Rubric methods as the later has *k*+1 interpretable dimensions due to it generates an additional dimension for the general factor

At the end of this process, it is possible to obtain an orthogonalized vector space in which some dimensions denote the concepts to be evaluated, and other dimension acts as a general factor. This is very similar to bifactor modeling as the concepts’ dimensions and the general factor do not share common variance. This is one of the main similarities of this method with bifactor models based on orthogonalization procedures such as the Schmid–Leiman orthogonalization (Schmid & Leiman, 1957; see also Reise, 2012; Reise et al., 2007; Rodriguez et al., 2016; Zhang et al., 2020). This procedure allows one to estimate the presence or absence of the assessment rubric’s concepts in constructed responses with a simple projection in that new meaningful space, but we can also estimate a general factor of common variance in the vector space. Figure 2 illustrates the ***β*** matrix of the bifactor Inbuilt Rubric method. As it can be seen, the dimensions of the Inbuilt Rubric method have *k* meaningful dimensions representing the target concepts (e.g., *k* = 5, *C*_1_–*C*_5_) and other *p*-*k* latent dimensions of the original semantic space (Fig. 2a). As previously stated, the dimensions of the original semantic space are added to matrix ***β*** using a standard basis (see also Hu et al., 2007, p. 414). In this study, the meaningful dimensions represent concepts like “Earth’s age,” “Lamarck,” “Darwin’s expedition,” “Darwin’s theory,” and “Transcendence” (see the description of the first instructional text for a more complete description of this example). On the contrary, the dimensions of the bifactor Inbuilt Rubric method have the same structure, except for one of the *p*-*k* latent dimensions of the original semantic space has been replaced by a general factor (Fig. 2b). In this specific case, there would be five meaningful dimensions in the semantic space that represent the target concepts (e.g., “Earth’s age,” “Lamarck,” “Darwin’s expedition,” “Darwin’s theory,” and “Transcendence”), an additional general factor that is supposed to represent the general knowledge common to these concepts (see the *G* dimension in Fig. 2b) and different latent dimensions of the original semantic space to preserve the dimensionality of the original semantic space.

The present study will test the reliability and consistency of the general and specific factors of the bifactor Inbuilt Rubric method. For this purpose, the scores of these versions of the Inbuilt Rubric method will be compared with the bifactor structure of human raters’ scores from a psychometric perspective.

### Using psychometrics to infer constructs from computational indicators

It is good practice in psychological research to empirically test the reliability and validity of psychological tests. This is why different psychometric approaches have been developed by means of classic theory tests, item response theory, or newer approaches (e.g., Abad et al., 2011; Maydeu-Olivares & McArdle, 2005; Raykov & Marcoulides, 2008; van der Linden & Hambleton, 2013). Thus, we need to provide evidence that every human or computational evaluation is valid. In classical psychometrics, testing the factor structure of observable variables lets researchers evaluate their hypothetical measurement models. In this way, researchers assume a model in which the observed indicators (e.g., test items) configure some unobserved constructs (such as academic skills). Factor analysis allows researchers to infer these constructs from the observed indicators when their models present a good fit to the data. Otherwise, researchers must review their indicators, models, or both. In psychoeducational tests, different statistical approaches, like structural equation models, can combine the measurement models of computational scores and the relations to be analyzed from a substantive point of view. We think that promoting the use of psychometrics to evaluate computational assessments is also a good practice. Specifically, the same scheme could be applied to observable measures derived from constructed responses; computational indicators (textual properties) are related to constructs, and such relations have an underlying measurement model.

Chapelle and Voss (2017) remarked that the technological advances in language testing and other natural language-processing evaluations need to show their comparability with other classic psychoeducational tests to improve the current approaches to language assessment (note a similar rationale behind how the term “validity” changed for language assessment in Chapelle, 1999). While there is an important relation between technological advances and language assessment (e.g., Chapelle & Voss, 2016, 2021), it needs to continuously improve the design of computer-assisted language tests and the ways to demonstrate their validity. In this regard, natural language processing (NLP) research and other advances in language testing systematically lack empirical tests of measurement models. Usually, single computational measures are used as predictors in NLP research as direct indicators of constructs. However, even in a more suitable scenario where different computational scores are added to generate a sum score, researchers would be losing statistical power due to strict constraints imposed on their underlying measurement models (e.g., McNeish & Wolf, 2020; Rhemtulla, 2016). Thus, finding evidence in favor of the underlying measurement model of the computational indicators is a way to not only validate the measures of an automatic system but also set the cornerstone of the measurement with important advantages compared with, for example, the sum of the scores due to their strict constraints (McNeish & Wolf, 2020). Furthermore, from a theoretical standpoint, it may not be possible to justify the use of computational scores to measure academic skills in the absence of a clear measurement model (e.g., the sum of computational scores could only be justified if there is an underlying unidimensional model or a similar factor structure). Among all the decisions that can be made to design psychoeducational assessment tasks involving automated scoring, Carr (2008) remarked that the most important one is to stay focused on the target constructs. Accordingly, measurement models can provide different validity evidence supporting the use of different computational methods, like Inbuilt Rubric, for evaluating various general and specific skills. As previously stated, to the best of our knowledge, only some works have recognized that it is mandatory to gain reliability and validity for the development of computational methods and other computer-mediated technologies (e.g., Attali, 2014; Bejar et al., 2016; Chapelle, 1999; Chapelle & Voss, 2021; Koskey & Shermis, 2014; Rupp, 2018).

### The present study

In summary, topics can be imposed a priori onto vector space models using techniques such as the Inbuilt Rubric method. It transforms the original vector space into a semantic space that maps the content of an assessment rubric designed by human raters. This method is used to create a system that identifies indicators from constructed responses and infers constructs of the student knowledge on these topics. In this regard, we aim to extend two different approaches for this method. First, we aim to validate a new hierarchical LSA vector space, generated using the bifactor Inbuilt Rubric method, to evaluate constructed responses in psychoeducational assessments. It is hypothesized that hierarchical models, like the bifactor Inbuilt Rubric, can increase the validity of computational assessments evaluating general and specific knowledge in vector space models. Thus, the general dimension in the vector space model is presumed to distill the semantic meaning of the specific concept dimensions. Second, we seek to evaluate a new approach that uses nouns embedded in the fragments of the instructional text, which is less demanding and more automatic than selecting lexical descriptors by consensus. For this purpose, two parallel processes of validation for the assessment of constructed responses will be conducted for the rubric assessments of human raters and the computational scores of the Inbuilt Rubric method. Both measurements were designed to fulfill the same task, and we will evaluate (a) whether they present similar factor structures (i.e., to evaluate the underlying measurement models, namely related factors and bifactor structure), and (b) the convergent and discriminant validity of computational scores from a substantive point of view. To do so, we aim to combine psychometrics, e.g., the use of measurement models, with the remarkable potential of computational assessment. It is known that computational methods can be affordable predictors of some behaviors or psychological phenomena. However, we need to provide empirical evidence for their validity. We think that promoting the use of psychometric techniques to evaluate computational assessments is a good practice for validating computational methods.

Thus, the objective of the present study is to validate the computational scores of different versions of the Inbuilt Rubric method, showing how common psychometric approaches can present different validity evidence for psychoeducational computational assessments. The specific objectives of this study are threefold: (1) to validate an alternative version of the Inbuilt Rubric method that does not require the selection of lexical descriptors (taking advantage of the vector representation of all the nouns within fragments of the instructional text), (2) to test whether the bifactor Inbuilt Rubric method is capable of increasing the convergent and discriminant validity of the computational scores as it distills the meaning of target concepts in the vector space, and (3) to show how psychometric measurement models can properly validate the automatic assessment of constructed responses. All these objectives are put to the test using human rater assessments as validity criteria. In the present study, we will analyze the construct validity of human rater scores using exploratory factor analyses (EFAs). Then, the human measurement models will be imposed onto the computational scores of the Inbuilt Rubric method using confirmatory factor analyses (CFAs). Both human and computational measurement methods were designed to fulfill the same topic-detection task, and they have the same hypothetical factor structures. Moreover, convergent and discriminant validity will be tested considering the measurement model of computational scores by means of SEM. A higher convergent and discriminant validity is expected using SEM and the bifactor Inbuilt Rubric as these procedures are supposed to distil the raw computational assessments of the target concepts. It is worth mentioning that, although this study was conducted in the Spanish language, the proposed scheme is language-independent and applicable to any language.

## Method

### Participants

A total of 205 Spanish undergraduate students (including 175 women; the average age was 20 years) took part in this study. They were tasked with summarizing three texts in approximately 250 words each (the mean length was 251 words per summary). Students were recruited voluntarily and received course credit for their participation in the present study. While the number of women was larger than that of men in the sample, no relevant differences were found between them concerning the length of their summaries or their performance (see the first section of “Results”). The open data set including student summaries and human rater evaluations are available in the OSF repository of this study.

### Instruments

#### Texts

Three Spanish expository texts were selected for the present study. The difficulty levels of the instructional texts were evaluated using two different criteria. First, they were evaluated according to the Spanish descriptors of each mastery skill from the Curriculum Plan of the Cervantes Institute (established by the Common European Framework of Reference for Languages, CEFR). Second, they were evaluated using different readability indices of Coh-Metrix-Esp (Quispesaravia et al., 2016), which is a Spanish adaptation of Coh-Metrix (Graesser et al., 2004, 2011; McNamara et al., 2014). The 45 readability indices of Coh-Metrix-Esp for each text are available in the OSF repository of this study.

- Text 1 is called *Darwin’s Theory of Evolution* (Asimov, 1969). This text is approximately 1300 words long and describes how Darwin was influenced by other authors and how he developed his theory of evolution. Its difficulty corresponds to level B2 in the CEFR.
- Text 2 is titled *Strangler Trees* (Peiro, 1972). This text is approximately 500 words long and discusses how species of trees compete for alimentary resources to survive. The text’s difficulty was level B1–B2 in the CEFR.
- Text 3, called *Language Evolution* (Martín-Loeches, 2016), is approximately 900 words long and presents different theories of the evolution of language. Its difficulty corresponded to level C1 in the CEFR.

In the Results section, it is noted that Text 1 and Text 3 did not present important differences in terms of their difficulty. However, the performance of Text 2 was higher than that of the other texts. This could be a substantive result, as Text 2 would be an easy instructional text for undergraduate students since it was originally designed for secondary education students as part of a standardized evaluation test (León et al., 2012).

#### Assessment rubrics

Assessment rubrics were designed by following inductive criteria. First, different human raters read the instructional texts and generated ideal summaries. These ideal summaries were then used to extract common and necessary topics from each instructional text by consensus (these conceptual axes have been previously validated in Martínez-Huertas et al., 2018, 2019, 2021). Thereafter, an assessment rubric with different conceptual axes was created for each instructional text. Each conceptual axis considered the inclusion of some sub-topics and a coherent discourse following the criteria established by Jonsson and Svingby (2007) and León et al. (2006). These scores of the conceptual axes ranged from 0 (indicating the absence of the target concept) to 2 (representing coherent and comprehensive explanation of the concept). For example, the evaluation of a conceptual axis (concept) of an instructional text would be as follows: a summary that does not mention the concept would receive a zero along the conceptual axis; a summary that gives a full explanation for it—summarizing all the relevant aspects of the original text—would receive the maximum score; and that which provides an incomplete or incoherent explanation of the concept would receive an intermediate score. The total score can be computed for each assessment rubric by adding all the scores of the conceptual axes. The conceptual axes (concepts) that should be included in good summaries were used to compound the following assessment rubrics:

- The rubric for Text 1 comprised five concepts: *Earth’s age* (with a maximum score of 2 points in the rubric), *Lamarck* (max = 2), *Darwin’s expedition* (max = 2), *Darwin’s theory* (max = 3), and *Transcendence* (max = 1).
- The rubric for Text 2 included four concepts: *Contextualization of the text* (max = 2), *Process of strangulation* (max = 2), *Competition between the trees for reaching sunlight* (max = 2), and *Strategy of survival* (max = 2).
- The rubric for Text 3 was composed of five concepts: *Debate* (max = 2), *Phonology* (max = 2), *Syntax* (max = 2), *Semantics* (max = 2), and *Symbol* (max = 2).

Note that two concepts, namely *Darwin’s theory* and *Transcendence* of Text 1, received a different score range due to their differential representativeness in the instructional text, but it did not compromise the results of the present study, as they are based on factor scores.

Dawson (2017) provided a synthesis of the diversity of rubrics to frame the instrument in each study. According to Dawson’s design elements, the assessment rubrics employed in this study would be task-specific (it assesses specific instances in particular course units), with an analytic scoring strategy (using individual criteria, combined to generate overall scores), evaluative criteria (distinguishing acceptable responses from unacceptable responses), and different levels of quality based on quality definitions (descriptors define the performance of individuals). Designed to conduct evaluation within experimental research, ensuring its reliability and validity, other characteristics of these rubrics are secrecy (the rubric was only shared with the participants after the evaluation) and high judgement complexity without exemplars or accompanying feedback information. We propose that the Inbuilt Rubric method scores could have the same characteristics (but both the human rater and computational scores could also be used to provide accompanying feedback information).

#### LSA´s semantic space

The initial linguistic corpus was composed of 455,969 documents (paragraphs) from a random sample of the Spanish Wikipedia. A total of 70,244 unique terms were processed to generate a latent semantic space. Log-entropy was used as the weighted function (see Nakov et al., 2001, for the use of this measure in LSA), and a total of 300 dimensions were imposed onto the latent semantic space following standard criteria (Evangelopoulos et al., 2012). This latent semantic space was later transformed using different versions of the Inbuilt Rubric method (the version with predefined lexical descriptors and the one with nouns embedded in the fragments of the instructional text). Both versions are generated by filling the first columns of matrix ***β*** with four or five meaningful vectors (representing sets of descriptors or fragments in Text 2 and Texts 1 and 3, respectively) and the remaining columns with 295 or 296 dimensions of the original latent semantic space to obtain the original dimensionality (in this case, 300 dimensions). Then, matrix ***β*** is orthogonalized via the Gram–Schmidt method to obtain a new basis. A correlation is calculated in the orthogonalization process to confirm that the orthogonalized meaningful vectors of ***β*** correlate with their non-orthogonalized version (0.80 or more was considered reliable). After this, a change of basis from the original latent standard basis to the basis represented by ***β*** is carried out (it is a simple rotation). The objective is to have all the words of the space (the term matrix) expressed in the ***β*** basis^6^. Afterward, the concepts of the constructed responses are identified and projected onto the new meaningful space. Gallito Studio software (Jorge-Botana et al., 2013) was used to implement both corpus training and the Inbuilt Rubric method. It is worth mentioning that the bifactor Inbuilt Rubric method incorporates an additional vector in the meaningful part of the ***β*** matrix. This additional vector is the vector denoting the general factor of a factor analysis with the descriptors or the nouns embedded in the fragments of the instructional text (see Jorge-Botana et al., 2019, for details).

### Procedure

Students were recruited and tasked with summarizing three expository texts. The order of presenting the instructional texts was randomized for each participant. Then, two human raters evaluated the summaries made by the students using the rubrics described in the Instruments section. Human assessments were performed before any computational evaluation, and both human raters independently rated the student summaries (blind assessment). These human rater evaluations are available in the OSF repository of this study (see the Open Practices Statement).

As mentioned, two different versions of the Inbuilt Rubric method were tested in the present study. The first version is the original one that involves transforming the latent semantic space using lexical descriptors predefined by human raters. Table 1 presents the lexical descriptors for each conceptual axis (these lexical descriptors were proposed by human raters who participated in other studies and were previously validated in Martínez-Huertas et al., 2018, 2019, 2021). Besides previous empirical validation, the quality of the lexical descriptors was evaluated by analyzing the semantic neighborhood of their vector representation, and usually three descriptors per concept are enough to accurately represent the concept for automated summary evaluation (Martínez-Huertas et al., 2018). The second version of Inbuilt Rubric is more automatic and does not require decision-making concerning the lexical descriptors. In this case, the latent semantic space was transformed using all the nouns embedded in the fragments of the instructional texts (see Fig. 1). This method is similar to other previous methods for content-detection tasks such as partial content similarity or partial golden summaries (Dessus & Lemaire, 1999; Franzke et al., 2005; Kintsch et al., 2007; Magliano & Graesser, 2012; Martínez-Huertas et al., 2021). This procedure results in a vector having different numbers of nouns for each concept to be evaluated. The latent semantic space was then transformed into a new meaningful one using both versions of the Inbuilt Rubric method (i.e., the version with predefined lexical descriptors and the one with nouns embedded in the fragments of the instructional text). In addition, a vector that represents a general factor was added to matrix ***β*** of both versions in the bifactor Inbuilt Rubric method. This means that we put to test four configurations of Inbuilt Rubric defined by the following dyads: descriptors/fragments and with/without general factor. All the student summaries and lexical descriptors were lemmatized before conducting the study.

**Table 1:** Lexical descriptors per text used to transform the latent semantic space

	Concepts	Lexical descriptors
Text 1	Earth’s age (C1)	Hutton Buffon earth
	Lamarck (C2)	Lamarck characteristics acquired
	Darwin’s expedition (C3)	Beagle Galapagos finches
	Darwin’s theory (C4)	selection natural evolution
	Transcendence (C5)	polemic biology modern
Text 2	Contextualization of the text (C1)	tree strangle Brasil
	Process of strangulation (C2)	kill asphyxiation roots
	Competition between the trees for reaching sunlight (C3)	competition lights sun
	Strategy of survival (C4)	adaptation survival survive
Text 3	Debate (C1)	Evolution Neuroscience Paleontology
	Phonology (C2)	Phonetics Articulation Deafness
	Syntax (C3)	Syntax Sentence Macromutation
	Semantics (C4)	Semantics Meaning Sign
	Symbol (C5)	Symbol Abstraction Flexibility

*Note.* Text 1 = *Darwin’s Theory of Evolution*. Text 2 = *Strangler Trees*. Text 3 = *Theory of the Evolution of Language*. C1-C5 = Concepts 1 to 5. Lexical descriptors were lemmatized before transforming the semantic space. These lexical descriptors were translated from Spanish

### Data analysis

Different statistical analyses were performed to present validity evidence for the scores given by human raters and different versions of the Inbuilt Rubric method. The analyses of the human rater scores were conducted to demonstrate the reliability and validity of the human evaluations, which are the dependent variables of the study. The analyses of the scores of different versions of the Inbuilt Rubric method were performed to show the validity of the raw computational scores. Subsequently, different approaches were used to test both the underlying measurement models of the computational scores and their convergent and discriminant validity predicting the human rater scores. All the statistical analysis were performed in R software (R Development Core Team, 2019).

First, the inter-rater reliability was estimated through intraclass correlation coefficients (ICCs) (Shrout & Fleiss, 1979), using a two-way mixed effects model in the psych package (Revelle, 2018). The inter-rater reliability was estimated for both the evaluation of concepts and the total scores of the rubrics of human raters. ICC results were assessed based on classic criteria (Cicchetti, 1994). Second, additional analyses were carried out to show the equivalence of the instructional texts. Different paired *t* tests and Cohen’s d were conducted to compare the mean length (number of words) of the student summaries between texts, and the same analyses were performed comparing the mean performance. These results were replicated in the comparison of women and men. Also, both dependent variables were related with the Pearson correlation coefficients. Third, Horn’s parallel analyses (scree plots) were conducted to retain the optimal number of factors of human rater scores using R’s psych package. The empirical eigenvalues (using principal components) were compared with those simulated via Monte Carlo simulation. We retained the factors whose empirical eigenvalues exceeded the 95th percentile of the simulated ones (PA_95_) (Glorfeld, 1995; Weng & Cheng, 2005). Fourth, once the number of factors was determined by parallel analyses (scree plots), EFAs were carried out using R’s psych package to analyze the factor structure of human rater scores. Maximum likelihood (ML) estimator and oblimin rotations were used to estimate these models to ease the interpretability of the factor loadings. These analyses were done for each text to validate the human rater scores, the validity criteria for computational scores in this study.

After the human assessments were done and validated, the computational scores were calculated, and different statistical analyses were performed to test their performance. In this way, different multiple linear regression models were estimated with R’s *lm* base function to analyze the convergent and discriminant validity of the raw Inbuilt Rubric method scores using human rater scores as criteria (human criteria were computed here as the factor score of EFAs). These results were used to validate the version of the Inbuilt Rubric method with nouns embedded in the fragments of the instructional text. Finally, SEMs were applied to test the convergent and discriminant validity of computational scores, considering their measurement models using the lavaan package (Rosseel, 2011). CFAs and SEMs were estimated using unweighted least squares^7^ (ULS), and standard cutoff criteria were applied to evaluate models’ fit to the data. Recommendations from Byrne (2012) were followed: first, we tested the measurement models of the scores given by the human raters and the two versions of the Inbuilt Rubric method using CFAs for each instructional text; second, we tested the whole SEM model for each instructional text. In this study, two different measurement models were used with human rater scores, namely correlated factors for concepts and the bifactor model. Inbuilt Rubric’s validity was evaluated using the correlated factors in human rater assessments, whereas the bifactor Inbuilt Rubric’s validity was evaluated with the bifactor model in human rater assessments. The model fit of all the factor analyses was assessed using standard criteria for χ^2^ tests.

## Results

### Validation of human rater scores

The following analyses were performed using the human rater assessments to present validity evidence in favor of their use as dependent variables of the study. Table 2 provides inter-rater reliability (ICCs) for human raters. According to classic criteria (Cicchetti, 1994), all ICCs are good to excellent (except for C4 of Text 3 that only obtained a moderate ICC). The most common use of these ICCs is to evaluate the total assessment scores, but we also wanted to show that the assessments of the concepts were reliable even when the variance of these scores was much smaller. Inter-rater reliability can be considered appropriate for both the total scores and different concepts of each instructional text. This result shows that the human rater assessments were reliable.

**Table 2:** Inter-rater reliability (intraclass correlation coefficients; ICCs) for each concept in the assessment rubrics

	C1	C2	C3	C4	C5	Total score
Text 1	.83	.64	.89	.65	.81	.85
Text 2	.63	.61	.64	.70	-	.69
Text 3	.67	.72	.66	.57	.81	.77

*Note.* All ICCs were statistically significant (*p*<.01). Reliability measures were established through ICCs using a two-way mixed effects model. Text 1 = *Darwin’s Theory of Evolution*. Text 2 = *Strangler Trees*. Text 3 = *Theory of the Evolution of Language*. C1–C5 = Concepts 1 to 5. C5 is not available for Text 2 as only four concepts were considered for it

Additional analyses were conducted to test potential differences in the results due to the different lengths of the instructional texts. Although the participants were asked to make summaries of 250 words, the length (number of words) of the student summaries was found to be different for each instructional text. The mean length per summary was 279 words (SD = 58.7) for Text 1, 213 words (SD = 49.5) for Text 2, and 261 words (SD = 59.2) for Text 3. The length of the summaries of Texts 1 and 3 was similar (*t*(204) = 4.86, *p* <.001, Cohen’s *d* = .34). On the contrary, Text 2 had significantly shorter summary lengths than Text 1 (*t*(204) = 15.41, *p* <.001, Cohen’s *d* = 1.08) and Text 3 (*t*(204) = 13.23, *p* <.001, Cohen’s *d* = .92). Similar results were found for the mean performance in each text. Mean performance was 1.16 (SD = .287) in Text 1, 1.40 (SD = .218) in Text 2, and 1.12 (SD = .253) in Text 3. The difficulty of Texts 1 and 3 was similar (*t*(204) = 2.08, *p* = .039, Cohen’s *d* = .145). Text 2 had significantly higher performance in comparison with Text 1 (*t*(204) = −10.30, *p* <.001, Cohen’s *d* = −.719) and Text 3 (*t*(204) = −14.64, *p* <.001, Cohen’s *d* = −1.022). The mean performance was higher for Text 2 because it was an easier instructional text (see the Instruments section), but this was not the case with summary length, as it had shorter student summaries. It was also found that the larger summaries tended to obtain higher total scores than the shorter ones (the Pearson correlation coefficient between the summary length and the total score was .59 for Text 1, .57 for Text 2, and .58 for Text 3).

Additionally, given that the number of women was larger than that of men in the sample, their differences were examined in terms of their summary length and performance. The mean number of words per summary by women was 278 (SD = 60.3) for Text 1, 215 (SD = 50.3) for Text 2, and 259 (SD = 56.8) for Text 3. The mean number of words per summary among men was 282 (SD = 49.2) for Text 1, 204 (SD = 44.1) for Text 2, and 275 (SD = 71.1) for Text 3. No relevant differences were observed between them in terms of Text 1 (*t*(203) = −.307, *p* = .759, Cohen’s *d* = −.06), Text 2 (*t*(203) = 1.072, *p* = .285, Cohen’s *d* = .211), or Text 3 (*t*(203) = −1.401, *p* = .163, Cohen’s *d* = −.276). Regarding their performance, the mean performance of women was 1.15 (SD = .292) for Text 1, 1.41 (SD = .207) for Text 2, and 1.12 (SD = .254) for Text 3. The mean performance of men was 1.20 (SD = .254) for Text 1, 1.33 (SD = .268) for Text 2, and 1.10 (SD = .252) for Text 3. No relevant differences were noted in their performance for Text 1 (*t*(203) = −.804, *p* = .422, Cohen’s *d* = −.158), Text 2 (*t*(203) = 1.849, *p* = .066, Cohen’s *d* = .365), or Text 3 (*t*(203) = .519, *p* = .605, Cohen’s *d* = .102). Thus, no relevant differences were found between their summary length or performance.

**Fig. 3:**
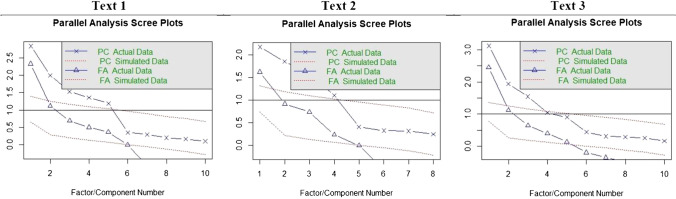
Parallel analysis (scree plots) for the scores of human raters in each text. *Note*: The comparisons of factor analysis (FA) actual data and factor analysis (FA) simulated data in scree plots revealed the underlying factor structures of human rater scores for each text

To show and validate the measurement model of the human rater assessments, a parallel analysis (scree plot) and an EFA were performed. The former analysis was conducted to extract the appropriate number of factors for the human rater assessments, while the latter was done to present the actual factor structure of the latent factors. Parallel analysis (scree plots) results for human rater scores are provided in Fig. 3. The number of underlying factors of human rater scores corresponds with the number of evaluated concepts in Text 1 and Text 2 according to the number of components and the number of factors of parallel analyses (that is, five and four factors were underlying their variance structure respectively). On the other hand, results for Text 3 showed a discrepancy: the number of underlying factors was five, and the number of components was three. In Text 3, three factors were retained using a more conservative criterion to avoid spurious factors/components.

Table 3 presents the standardized factor loadings (pattern matrix) for EFAs of human rater scores. A good factor structure was found with regard to the concepts of the instructional text (which is in accordance with the hypothetical factors suggested by the design of the measurement model). The fit of the models was good for Text 1 (root mean square error of approximation—RMSEA [90% confidence interval—CI] = .033 [.000–107], Tucker–Lewis index—TLI = .99, root mean square of residuals RMSR = .01), and Text 2 (RMSEA [90% CI] = .061 [.000–.164], TLI = .957). On the contrary, the model fit for Text 3 was poor (RMSEA [90% CI] = .197 [.170–.226], TLI = .549). These analyses were interpreted as a description of the factor structure of the measures. The standardized factor loadings show that the concept evaluations by the human raters have sufficient factorial validity.

**Table 3:** Standardized factor loadings (OBLIMIN rotation) for exploratory factor analyses of the scores of human raters in each text

		Text 1					Text 2				Text 3		
Variables		F1	F2	F3	F4	F5	F1	F2	F3	F4	F1	F2	F3
HR1	C1	.83	.00	.08	−.02	.03	.64	.02	−.02	−.06	.99	.06	.03
	C2	−.02	1.00	−.02	.03	.03	.12	.71	.03	.02	−.04	.17	−.04
	C3	.03	.02	.90	−.03	.02	.00	−.03	1.00	.02	.03	.96	.14
	C4	−.10	−.01	.04	.85	−.01	.00	−.01	.01	1.00	.06	.21	.28
	C5	.00	−.02	.01	.00	1.00	–	–	–	–	.08	.20	.78
HR2	C1	.98	.00	−.04	.02	−.01	1.00	.00	.00	.01	.68	.04	.08
	C2	.05	.64	.07	−.10	−.12	−.06	.87	−.01	.00	.00	.14	−.05
	C3	−.02	−.02	.99	.03	−.01	−.01	.08	.72	−.06	.08	.63	.15
	C4	.14	.02	−.02	.80	.01	−.01	.06	−.04	.69	.10	.08	.04
	C5	.00	.01	.00	.00	1.00	–	–	–	–	.06	.11	.99

*Note*: HR1–HR2 = Human raters 1 and 2. C1–C5 = Evaluation of concepts of each instructional text. F1–F5 = Empirical factors for each text. C5 is not available for Text 2 as only four concepts were considered for it

### Multiple linear regressions to evaluate the raw scores of the Inbuilt Rubric method

In this section, we tested the convergent and discriminant validity of the raw computational scores obtained by the Inbuilt Rubric method^8^. For this purpose, a multiple linear regression was computed for each concept. Here, the mean human rater assessment for each concept was used as a dependent variable (HR C1–HR C5), and the performance of the different meaningful dimension scores of the Inbuilt Rubric method were included as covariates and evaluated through the standardized β coefficients (*β*_1_–*β*_5_). Table 4 lists the standardized *β* coefficients of different multiple linear regressions predicting the scores by human raters using the computational scores. Good convergent and discriminant validity was observed for both versions of Inbuilt Rubric in Text 1 and Text 2. It is worth mentioning here that, although they correct measure the target concepts, some standardized *β* coefficients are not very high (e.g., standardized *β* = .06). In Text 3, the first human concept (HR C1) was mainly measured by the first dimension (*β*_1_), whereas the third human concept (HR C3) was mainly measured by the last dimension (*β*_5_). The rest of the dimensions (*β*_2_, *β*_3_, *β*_4_) measured the second human concept (HR C2). These results are useful to validate and show the equivalence of the performance of the Inbuilt Rubric method with nouns embedded in the fragments of the instructional text, compared with the classic Inbuilt Rubric.

**Table 4:** Standardized β coefficients from multiple linear regressions to detect concepts using raw scores of two different versions of the Inbuilt Rubric (IR) method

			Standardized β coefficients					R^2^
			*β*_1_	*β*_2_	*β*_3_	*β*_4_	*β*_5_	
Text 1	IR1	HR C1	.37^**^	−.15^*^	.07	.01	.14^*^	.21
		HR C2	.02	.23^**^	−.05	−.10	−.01	.05
		HR C3	−.06	−.15	.31^**^	−.11	−.12	.15
		HR C4	−.10	−.03	.08	.20^*^	−.09	.06
		HR C5	.08	−.08	.05	.21^**^	.35^**^	.21
	IR2	HR C1	.49^**^	−.14^*^	.11	.07	−.04	.30
		HR C2	.07	.28^**^	.11	−.05	−.04	.08
		HR C3	−.10	−.12^*^	.54^**^	−.01	−.18^**^	.34
		HR C4	−.12	−.18^**^	.15^*^	.40^**^	−.14^*^	
		HR C5	.07	−.10	.01	.13	.34^**^	.18
Text 2	IR1	HR C1	.45^**^	−.16^*^	.04	−.15^*^	.22	
		HR C2	−.03	.38^**^	.07	−.24^**^	.16	
		HR C3	−.15^*^	−.03	.14^*^	−.03	.05	
		HR C4	−.04	.02	.06	.23^**^	.06	
	IR2	HR C1	.40^**^	−.06	−.10	−.14^*^	.21	
		HR C2	−.02	.33^**^	.06	−.20^**^	.13	
		HR C3	−.08	−.08	**.06**	−.03	.02	
		HR C4	−.04	.10	−.02	.20^**^	.06	
Text 3	IR1	HR C1	.31^**^	−.05	.03	.01	.05	.10
		HR C2	−.19^**^	.28^**^	.10	.13	−.03	.21
		HR C3	.02	.11	−.16	−.09	.34^**^	.18
	IR2	HR C1	**.09**	−.07	−.02	.05	.03	.02
		HR C2	.05	.39^**^	.12	.02	−.10	.22
		HR C3	.11	−.06	−.02	−.05	.41^**^	.21

*Note.* IR1 = Inbuilt Rubric method with lexical descriptors. IR2 = Inbuilt Rubric method with nouns embedded in fragments of the instructional text. HR C1–HR C5 = Human rater concept scores. HR C5 is not available for Text 2 as only four concepts were considered for it. ** = *p*<.01. * = *p*<.05. In bold = Best predictions (largest standardized β coefficients). Dependent variables were estimated factor scores of EFAs

### Structural equation models to evaluate the scores of the Inbuilt Rubric method

Previous section showed the convergent and discriminant validity of the raw computational scores of the two versions of the Inbuilt Rubric method. In this section, we examine whether it is possible to improve the performance of these computational scores considering their measurement models by means of SEMs. SEMs are a multivariate statistical technique that allows one to analyze multiple and interrelated dependencies between the measured constructs in their structural part. In this study, such multiple and interrelated dependencies were used to evaluate the convergent and discriminant validity of the Inbuilt Rubric method scores, taking into account their measurement models. Here, the endogenous factors were measured by the human rater assessments (HR C1–HR C5), and the exogenous ones were measured by the Inbuilt Rubric method scores (IR C1–IR C5). Recommendations from Byrne (2012) were followed to test these SEMs. First, the measurement models of the human raters and the Inbuilt Rubric method scores were tested using CFAs. Second, the convergent and discriminant validity of the computational scores was evaluated with SEM by estimating all the cross-loading parameters between the Inbuilt Rubric and human rater concept factors.

Specifically, measurement models were tested with CFAs for human raters and the Inbuilt Rubric method scores in each text. Table 5 presents the model fit of CFAs. As expected from the EFA results, model fits for all human rater scores were excellent. Also, model fits for the Inbuilt Rubric method scores were good. Text 3 had a relatively worse model fit than that of the other instructional texts for both human rater and computational scores.

**Table 5:** Confirmatory factor analyses (CFAs) of human raters and Inbuilt Rubric method scores for instructional texts

Text	Scores	χ^2^	*df*	CFI	TLI	RMSEA [90% CI]	SRMR
Text 1	Human raters	17.284	30	1.00	1.00	.000 [.000–.000]	.039
	Inbuilt Rubric	84.72	30	.925	.887	.095 [.071–.119]	.087
Text 2	Human raters	7.842	18	1.00	1.00	.000 [.000–.000]	.033
	Inbuilt Rubric	34.738	18	.975	.962	.068 [.032–.101]	.069
Text 3	Human raters	145.436	34	.860	.815	.127 [.106–.148]	.114
	Inbuilt Rubric	138.359	34	.904	.873	.123 [.102–.144]	.111

*Note*. Measurement models were fitted with ULS estimator

**Fig. 4:**
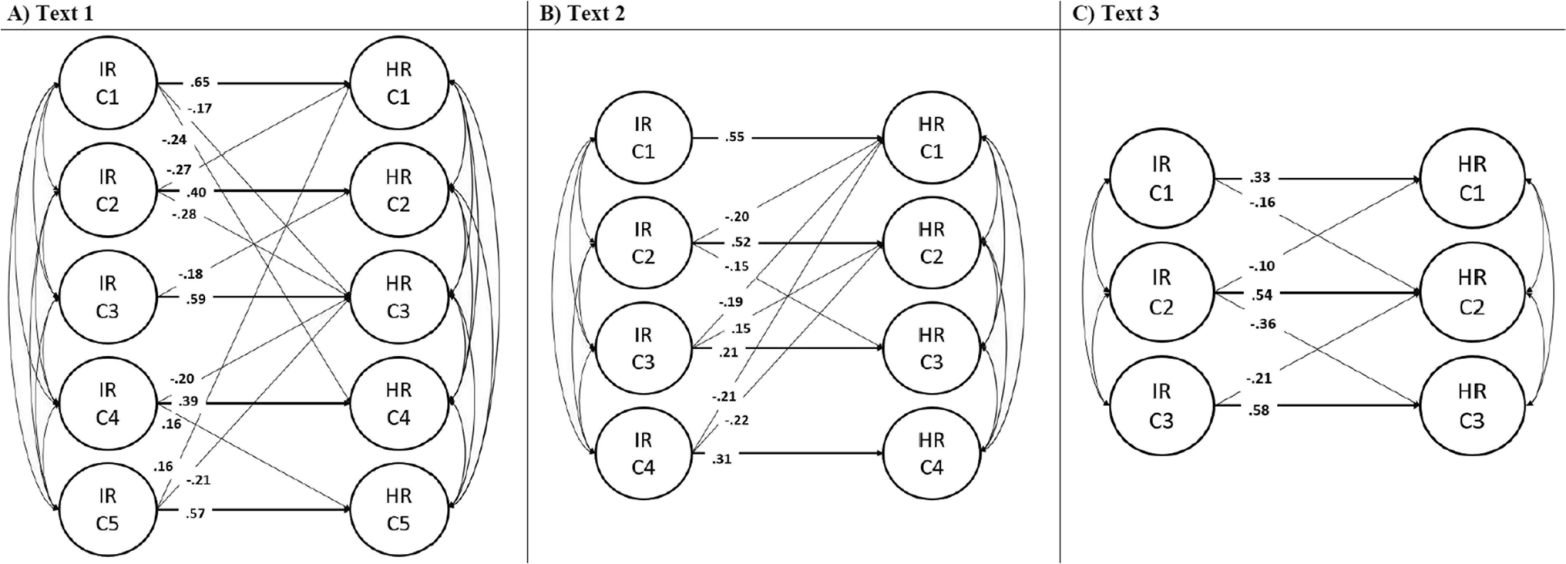
Structural equation model (SEM) results (standardized solution) for each instructional text. **a** Text 1, **b** Text 2, **c** Text 3. *Note*: Only the structural part of the SEM is reported in the figure. IR C1–IR C5 = Inbuilt Rubric concept factors. HR C1–HR C5 = Human rater concept factors. A full cross-loading model was estimated in each text, but the graph represents the statistically significant loading weights (*p*<.05), and bold lines represent the highest loading weight per factor

Then, a SEM was fitted to analyze the convergent and discriminant validity of the Inbuilt Rubric method scores for each instructional text. A good model fit was found for Text 1 (χ^2^(135) = 186.206, CFI = .976, TLI = .967, RMSEA [90% CI] = .043 [.026–.058], SRMR = .066), Text 2 (χ^2^(84) = 65.427, CFI = 1.00, TLI = 1.00, RMSEA [90% CI] = .000 [.000–.011], SRMR = .049), and Text 3 (χ^2^(159) = 452.593, CFI = .876, TLI = .852, RMSEA [90% CI] = .095 [.085–.105], SRMR = .103). Figure 4 shows the standardized results regarding the structural part of each SEM. As it can be seen, the convergent and discriminant validity was very good across all instructional texts (that is, the highest regression weights were the expected ones, and the other cross-loadings were not large). Moreover, an increase in effect sizes was observed for some concepts compared with the results of raw computational scores.

### Structural equation models to evaluate the scores of the bifactor Inbuilt Rubric method

The previous section tested the convergent and discriminant validity of computational scores of the two versions of Inbuilt the Rubric method within a latent framework. In this section, we examine the convergent and discriminant validity of the computational scores of the bifactor Inbuilt Rubric method. While the measurement model of the classic Inbuilt Rubric method has a first-order factor structure, the measurement model of the bifactor Inbuilt Rubric method has a bifactor structure. Again, the endogenous factors were measured by the human rater assessments (HR C1–HR C5), and the exogenous ones were measured by the Inbuilt Rubric method scores (IR C1–IR C5). The observed measures or indicators load onto a general factor representing their common variance, and the correlations between specific factors were imposed to be zero. We also followed the recommendations from Byrne (2012) for testing SEMs. First, we tested the bifactor structure for human rater scores. Then, we generated a SEM for each text where we included the latent factors of the specific factors and the observed variables of the general factor of this version of Inbuilt Rubric. In this model, the observed general scores of the bifactor Inbuilt Rubric method were used to predict the general latent factor of the human rater scores, and a covariance parameter was included between the general scores of the computational scores. Thus, we evaluated the convergent and discriminant validity of the bifactor Inbuilt Rubric method scores with SEM by estimating all the cross-loading parameters between the bifactor Inbuilt Rubric and human rater concept factors and adding paths to connect the observed measures or indicators of the general factor of the bifactor Inbuilt Rubric methods (IR1G, IR2G) with the general latent factor of the human rater assessments (here, HRG).

The bifactor measurement models achieved appropriate model fit for Text 1 (χ^2^(39) = 93.972, CFI = .928, TLI = .917, RMSEA [90% CI] = .083 [.062–.105], SRMR = .092), Text 2 (χ^2^(23) = 49.575, CFI = .936, TLI = .922, RMSEA [90% CI] = .075 [.046–.104], SRMR = .082), and Text 3 (χ^2^(32) = 77.531, CFI = .943, TLI = .920, RMSEA [90% CI] = .084 [.060–.107], SRMR = .083). A SEM was fitted to analyze the convergent and discriminant validity of the Inbuilt Rubric method scores for each instructional text. A good model fit was found for Text 1 (χ^2^(182) = 452.178, CFI = .893, TLI = .864, RMSEA [90% CI] = .085 [.075–.095], SRMR = .094), Text 2 (χ^2^(117) = 255.762, CFI = .931, TLI = .910, RMSEA [90% CI] = .076 [.064–.089], SRMR = .086), and Text 3 (χ^2^(197) = 537.189, CFI = .883, TLI = .863, RMSEA [90% CI] = .092 [.083–.101], SRMR = .102). Table 6 presents the results concerning the structural part of each SEM. The convergent and discriminant validity was excellent across all instructional texts (that is, the highest regression weights were the expected ones). An increase in effect sizes can be observed for some concepts^9^.

**Table 6:** Structural equation model (SEM) results (standardized solution) of the structural parameters for each instructional text using the bifactor Inbuilt Rubric method

Parameter	Text 1				Text 2				Text 3			
	Estimate	*SE*	z-value	Std. Estimate	Estimate	*SE*	z-value	Std. Estimate	Estimate	*SE*	z-value	Std. Estimate
IR1~HR C1	**.629**	**.076**	8.27^**^	**.605**	**.666**	**.095**	7.04^**^	**.545**	**.318**	**.066**	4.80^**^	**.372**
IR2~HR C1	−.089	.064	−1.38	−.075	1.173	.165	7.13^**^	.529	−.170	.057	−2.98^**^	−.142
IR3~HR C1	−.092	.041	−2.23^*^	−.093	−.164	.058	−2.84^**^	−.143	.182	.065	2.81^**^	.179
IR4~HR C1	−.333	.080	−4.17^**^	−.253	−.045	.056	−.80	−.038	–	–	–	–
IR5~HR C1	.153	.054	2.81^**^	.129	–	–	–	–	–	–	–	–
IR1~HR C2	−.243	.048	−5.08^**^	−.264	−.074	.060	−1.23	−.062	−.062	.032	−1.96	−.070
IR2~HR C2	**.258**	**.072**	3.56^**^	**.247**	**1.890**	**.297**	6.37^**^	**.872**	**.527**	**.048**	10.87^**^	**.422**
IR3~HR C2	−.268	.045	−6.01^**^	−.307	−.121	.056	−2.13^*^	−.108	−.122	.040	−3.08^**^	−.115
IR4~HR C2	.188	.071	2.66^**^	.161	.100	.057	1.73	.087	–	–	–	–
IR5~HR C2	.158	.053	3.00^**^	.150	–	–	–	–	–	–	–	–
IR1~HR C3	.100	.045	2.21^*^	.106	−.174	.056	−3.11^**^	−.194	.037	.055	.675	.034
IR2~HR C3	−.104	.065	−1.61	−.097	.140	.057	2.45^*^	.166	.080	.059	1.36	.053
IR3~HR C3	**.537**	**.063**	8.58^**^	**.600**	**.700**	**.113**	6.21^**^	**.430**	**.894**	**.207**	4.31^**^	**.697**
IR4~HR C3	.303	.078	3.91^**^	.253	.106	.056	1.90	.123	–	–	–	–
IR5~HR C3	.170	.055	3.11^**^	.158	–	–	–	–	–	–	–	–
IR1~HR C4	−.041	.045	−.902	−.037	−.377	.064	−5.89^**^	−.287	–	–	–	–
IR2~HR C4	−.093	.065	−1.44	−.075	−.003	.092	−.04	−.001	–	–	–	–
IR3~HR C4	−.104	.042	−2.47^*^	−.100	.024	.054	.43	.019	–	–	–	–
IR4~HR C4	**.675**	**.118**	5.71^**^	**.486**	**.380**	**.073**	5.25^**^	**.302**	–	–	–	–
IR5~HR C4	.492	.069	7.74^**^	.393	–	–	–	–	–	–	–	–
IR1~HR C5	.197	.048	4.11^**^	.202	–	–	–	–	–	–	–	–
IR2~HR C5	−.105	.065	−1.63	−.096	–	–	–	–	–	–	–	–
IR3~HR C5	−.192	.044	−4.41^**^	−.209	–	–	–	–	–	–	–	–
IR4~HR C5	−.003	.072	−.04	−.002	–	–	–	–	–	–	–	–
IR5~HR C5	**.825**	**.103**	8.00^**^	**.740**	–	–	–	–	–	–	–	–
IR1G~HRG	.826	.203	4.08^**^	.284	1.144	.363	3.15^**^	.326	1.665	.456	3.65^**^	.398
IR2G~HRG	−.108	.188	−.57	−.037	1.540	.404	3.81^**^	.439	−.712	.0286	−2.49^*^	−.170
IR1G~~IR2G	−.312	.072	−4.31^**^	−.326	.485	.088	5.50^**^	.571	−.134	.074	−1.798	−.148

#### *Note*.

 IR1–IR5 = Bifactor Inbuilt Rubric concept factors. HR C1–HR C5 = Human rater concept factors. IR1G = General factor of bifactor Inbuilt Rubric with lexical descriptors. IR2G = General factor of bifactor Inbuilt Rubric with nouns embedded in fragments of the instructional text. HRG = Human rater general (bifactor) factor. HR C5 is not available for Text 2 as only four concepts were considered for it. HR C4 and HR C5 is not available for Text 3 as only three concepts were considered for it. ** = *p* < .01. * = *p* < .05. In bold = Best predictions (largest standardized factor loadings)

## Discussion

There is an important relation between technological advances and language assessment, but it needs to continually improve the design of computer-assisted language tests and the ways to demonstrate their validity. A promising proposal is to make the technological advances in language testing and other natural language-processing tasks comparable to classic psychoeducational tests (see, for example, different rationales behind how the term “validity” have changed for language assessment scheme: Chapelle, 1999; Chapelle & Voss, 2017). In this line, the automatic assessment of constructed responses can be useful to infer different constructs from a big set of indicators (e.g., Foltz et al., 2013). It also can have different levels of analysis supported by highly complex predictive models. For instance, in the case of topic detection, good performance has been achieved with supervised models in different tasks (e.g., Hashimoto et al., 2016; Lee et al., 2006; Li et al., 2015; Li et al., 2016b, b). However, supervised models cannot be implemented without having training sets of hundreds or thousands of pre-scored samples, and this is a time-demanding task (Dronen et al., 2015). In this regard, the main advantage of the Inbuilt Rubric method is that it does not need such pre-scored samples of constructed responses (Jorge-Botana et al., 2019; Martínez-Huertas et al., 2018, 2019, 2021; Olmos et al., 2014, 2016). In this method, a rational expert criterion (here, an assessment rubric in psychoeducational assessment) is imposed onto the vector space. For this reason, it is cheaper and more versatile than supervised models. It is cheaper since it is not time-demanding and versatile since rubric designers can change the concepts and descriptors of the rubric. In addition, the Inbuilt Rubric is well suited for feedback systems in which part of the feedback information could be a function of the scores in each concept dimension of the vector space. For these reasons, it enables early deployment with further refinements.

Nonetheless, it is suggested to test the Inbuilt Rubric configurations to gather validity evidence to ensure that these non-supervised implementations are consistent. Accordingly, one aim of this study was to use a standard psychometric approach like SEM to validate the assessments of the Inbuilt Rubric method by testing their measurement models and performance within a latent framework. Good convergent and discriminant validity evidence with human rater scores was observed for these computational assessments. It was also noted that the factor structures of the scores given by human raters and the Inbuilt Rubric method were equivalent. This is a very important construct validity evidence for computational scores. In fact, the measurement models of computational scores were found to improve the convergent and discriminant validity of the raw computational scores by means of SEM (as the raw computational scores lead to attenuated relationships by not adjusting for measurement errors). While different validity evidence, like fitting the underlying factor structure, is usually required to verify the scores of psychological tests, many NLP psychoeducational research does not consider their measurement models. In fact, to the best of our knowledge, it is not very common to empirically test them when using computational scores. In this way, much NLP research use computational measures as direct indicators of constructs imposing strict concerns on them as they do not consider their factor structure. This study exemplifies the potential of classic psychometrics to sort computational scores within a coherent frame with observable properties and inferred skills. The direct consequence is that we can jointly obtain reliability and validity evidence, with the latter being one of the most important objectives in validating computational assessments (Attali, 2014; Bejar et al., 2016; Koskey & Shermis, 2014; Rupp, 2018). Testing the measurement models in relation to empirical data allows one to obtain guarantees about the constructs that are intended to be measured. Even more importantly, they can improve methods’ performance for further deployments.

Another aim of the present work was to validate an alternative version of the Inbuilt Rubric method that uses nouns embedded in fragments of instructional text. This alternative procedure does not require the selection of lexical descriptors, thus avoiding a very systematic and thorough task wherein lexical descriptors are established by consensus. It was found that nouns embedded in the fragments of the instructional text can be an affordable alternative to use in the Inbuilt Rubric method when one wants to avoid decisions about the selection of lexical descriptors by consensus (it is a more automatic procedure). Selecting text fragments that represent the target concepts to be evaluated generates different possibilities, including using text fragments or definitions of concepts, that should be evaluated in future research. One limitation of this study is that, while there are many other potential ways of using the Inbuilt Rubric method, only text fragments were used to illustrate it. In any case, these text fragments can be part of the instructional text or other educational materials, allowing the generation of meaningful semantic spaces using more complex information than lexical descriptors. In this regard, these findings could complement extractive summarization in the future. Given that fragments from the source text are automatically extracted using some crucial parameters (Ozsoy et al., 2011; Steinberger & Jezek, 2004), the Inbuilt Rubric method could determine whether, for example, the selected fragments are important and sufficiently non-redundant (Kireyev, 2008; Vargas-Campos & Alva-Manchego, 2016). This would be a fully automatic procedure to use in this computational method. In this vein, the present study only used nouns as representative information conveying the semantic context of the target concepts within expository texts. However, other types of information (e.g., concerning verbs, adverbs, determinants) can also be used to transform the vector space. A clear example of the importance of verbs and similar types of information for construct representations within narratives, among others, is the event-indexing model (Zwaan et al., 1995). The evidence in favor of this model showed how multilevel and multidimensional memory representations of narratives are indexed based on time, space, protagonist, causality, and intentionality (Zwaan et al., 1995). Transforming vector spaces using these multidimensional models opens the door for future research into the representations of different types of texts, like narrative texts or natural language conversations, from a computational point of view. In fact, verbal and other types of measures already play a crucial role in systems such as Coh-Metrix to automatically score texts and essays (Graesser et al., 2011), with verbs being an especially relevant indicator of text difficulty (McNamara et al., 2012, 2014). Thus, while nouns embedded in fragments of instructional text seem to be an affordable means of assessing expository texts using the Inbuilt Rubric method, it is worth examining other substantive approaches from a theoretical perspective.

A third aim was to test whether the bifactor Inbuilt Rubric method (Jorge-Botana et al., 2019) could increase the convergent and discriminant validity of the computational scores with a general knowledge factor in the vector space. Results showed that this general factor can distill the common variance of the concepts of the vector space. Thus, the bifactor Inbuilt Rubric method is well suited for the assessment of general knowledge and could increase the validity of these computational scores. It presented higher convergent and discriminant validity than the raw computational scores and the original Inbuilt Rubric method in some concepts. In this context, imposing a general factor in the vector space increased the “distillation” of specific scores. It seems that the use of hierarchical models, such as bifactor models (Reise, 2012; Reise et al., 2007; Rodriguez et al., 2016; Zhang et al., 2020), could generate honed vector space models by means of the general knowledge factors. In any case, further research is needed on the interpretation of general knowledge factors in vector spaces, as the actual relation between the general factor of the bifactor Inbuilt Rubric and the general factor of the human raters was dependent on the version of the Inbuilt Rubric method and the instructional text. All these conclusions are directly related to psychoeducational assessments of constructed responses. However, such general dimensions may provide substantive variance to distill the modeling of other cognitive processes working as a proxy of general semantic noise to distill compositional processes (e.g., Günther & Marelli, 2020; Marelli et al., 2017) or modulate similarity judgments of concepts (e.g., Ichien et al., 2021; Netisopakul et al., 2021).

Various studies have tried to interpret the meaning of the vector space dimensions from an exploratory means (Evangelopoulos, 2013; Evangelopoulos et al., 2012; Evangelopoulos & Visinescu, 2012; Kallens & Dale, 2018; Kundu et al., 2015; Visinescu & Evangelopoulos, 2014). Their approaches are interesting in promoting the use of meaningful scores from the vector space, but they are qualitatively different from the Inbuilt Rubric method, as the latter imposes the meaning of concepts onto the vector space a priori. Of course, the performance of these methods could be enhanced by machine learning approaches such as neural networks or support vector machines. In fact, machine learning and other algorithms are improving current educational schemes in different ways (e.g., Alenezi & Faisal, 2020; Vaishnavi & Ravichandran, 2021; Zhai, 2021). For example, one of the most popular psychoeducational technologies is AutoTutor, an intelligent tutoring system (Graesser et al., 1999). This system has been evolving over the past decade by the implementation of multiple learning resources (e.g., see ElectronixTutor by Graesser et al., 2018). Thus, machine learning and other algorithms can handle different computational assessments to generate fine-grained scores for the assessment of constructed responses. In this paper, we tried to promote the use of computational scores, like that of vector space models, for the assessment of constructed responses, considering their validity from a psychometric standpoint. This means that different psychometric properties, such as the measurement model of computational scores, should also be evaluated prior to their use in psychoeducational assessments regardless of whether they are used as direct indicators of constructs or as a part of a machine learning-based algorithm. While this study aimed to validate a method focused on the detection of semantic concepts to promote the use of meaningful semantic spaces, we would like to note that these computational methods have the potential to complement other higher-order intelligent systems for improving the evaluation of different target concepts. This is because the main facet of the Inbuilt Rubric method and other similar procedures is the validity-centered approach of its multi-vector representations. As shown in the present study, such multi-vector representations can provide useful information for psychoeducational assessments using meaningful semantic spaces with or without general factors in the hierarchical vector space.

Although the different distributional semantic models could be conceived as different parameterizations with the same capacity to model cognitive processes (e.g., Günther et al., 2019; Jones et al., 2018; or Jorge-Botana et al., 2020), future research should aim to validate the Inbuilt Rubric and the bifactor Inbuilt Rubric methods in other vector space models, as it has only been validated in the LSA. This opens the door for examining whether it is possible to impose concepts a priori without mandatory orthogonal vector spaces like that of the popular Google *word2vec* (e.g., Mikolov et al., 2013). Future studies should analyze the differences that could be expected between vector space models regarding both the distillation of their scores and their measurement models. The dimensions of oblique vector spaces, like the word2vec model, could have large covariances, and the general factor could thus capture a large part of substantive variance. The dimensions of orthogonal vector spaces, like those of the LSA model, do not covary, and they are expected to partially reduce such a problem. Thus far, the generation of matrix ***β*** would be the same in both types of vector space models where the resulting vector space is expected to retain the semantic properties of the original vector space even with oblique dimensions. This would be translated into a differential performance of the general factor depending on the properties of the original vector space.

Another limitation of this study is the lack of model fit for the ML estimator in the models of computational scores. While the models for human rater scores achieved appropriate model fit using the ML estimator, the models for computational scores presented multiple convergence problems when they were fitted with ML. The ULS estimator did not produce relevant differences in terms of convergence. The ML estimation method occasionally leads to convergence problems when there are several local maxima in the log-likelihood function. This is likely to occur in Pearson correlation matrices from computational methods due to their orthogonal nature. Differences between the ML and OLS methods have been associated with weak common factors, and the latter is recommended when relevant differences are found (e.g., Briggs & MacCallum, 2003). Future research should investigate the performance of common estimators using different types of computational measures.

Also, it should be noted that the present study is just an illustration of the potential of the Inbuilt Rubric and bifactor Inbuilt Rubric methods in a specific educational setting. Such an illustration was made with a sample of undergraduate students who summarized three different instructional texts covering academic topics. First, only undergraduate students participated in this study. Given that the general scores of the original Inbuilt Rubric method could discriminate between different educational levels (Martínez-Huertas et al., 2019), it would have been interesting to test whether the general factor of the bifactor Inbuilt Rubric method could have different meanings in different educational levels. For example, such a general factor could reflect general knowledge in higher educational levels and a lack of knowledge in lower educational levels. Future research should experimentally test which variables affect the meaning of the general dimension. Second, only three instructional texts were used to illustrate the performance of these computational methods. While all the participants summarized the three instructional texts to gain internal validity, these computational scores were validated in an artificial educational setting, so future research should evaluate them in ecological contexts.

### Conclusion

One of the main contributions of this study is that it showed how standard psychometric procedures can validate and hone computational psychoeducational assessments. This creates an opportunity to fully combine computational semantics and standard psychometrics. This approach could increase the performance of the current measurement approaches using computational semantic measures to study their relations with different psychological constructs. Future research should test other potential advantages of the combination of computational methods and psychometrics from a validity-centered approach. One of our predictions is that hierarchical models, such as bifactor models, could generate important shifts in the use of computational scores from a theoretical and a methodological point of view. Our findings using the bifactor Inbuilt Rubric method, which is a hierarchical vector space, support such conclusions and further show that there is room for improvement in the current automatic assessments of constructed responses.

## Footnotes

1 R code, the semantic space, the student summaries, and the human raters’ assessments can be accessed via the following link: https://osf.io/m6akh/?view_only=f835c46896834c5abcdb0c66e9a6ba22. An R function has been developed to apply the different versions of the IR method (classic/bifactor and sequential/non-sequential; see R code). It can be used with lexical descriptors and/or nouns embedded in fragments of instructional text. Thus, R code can generate the IR method in its classic and hierarchical (bifactor) forms. Also, the Spanish students’ summaries and human raters’ evaluations can be used in other studies focused on human and automatic assessments of constructed responses (student summaries) in Spanish.

2 There are methods based on supervised learning algorithms using the vector representation from Bag of Words or dimensionality reduced vectors as input, but they require a reasonable large sample of text responses evaluated by human raters to train the model. For this reason, these methods are time-demanding and thus more expensive.

3 There is a discipline called *computational psychometrics* that is, basically, a prediction-centered approach. von Davier (2015) defined it as a mix of data-driven computer science methods (such as machine learning) that are focused on the scoring of real-time abilities. Moreover, *computational psychometrics* does not usually apply computational models of language. Thus, this approach is significantly different from the one that we propose here, namely the combination of computational semantics and psychometrics with a validity-centered approach.

4 The term “latent meaning” indicates that the semantic space and its vector representations do not have directly interpretable meanings.

5 Nouns were chosen here as an accurate representation of the semantic context of the target concepts to be evaluated. This is because the two versions of the Inbuilt Rubric method aim to generate the same semantic context (the one wherein the target concepts appear). As the present study was conducted using three expository texts involving very concrete and specific concepts, the semantic context of the concepts was basically determined by the nouns (not the verbs, which were common and unspecific). For example, a representative sentence of Text 1 is “*En 1809 presentó Lamarck su teoría de la evolución en un libro titulado Filosofía Zoológica.*” [“In 1809, Lamarck presented his theory of evolution in a book entitled 'Zoological Philosophy’”]. This semantic context is basically defined by nouns and seems to be sufficient for the assessment of concepts. Thus, while there are theoretical perspectives that highlight the role of information different from that of nouns (e.g., verbal information), we think that it would be more useful to explain ecological phenomena in narrative texts or texts with natural language. Future research has been proposed in the Discussion section about the use of different types of information (e.g., related to verbs, adverbs, determinants, etc.) to transform vector spaces.

6 Notably, the dimensions of the original semantic space present different properties—for example, their eigenvalues. Thus, the R script of the Inbuilt Rubric method has the possibility of implementing it sequentially or non-sequentially. In the first scenario, the dimensions of matrix ***β*** are filled sequentially, that is, from the first to the *kth* dimension. In the second scenario, the *k* dimensions of matrix ***β*** are filled non-sequentially; in other words, they are randomly ordered, and a final average ***β*** matrix is computed to avoid potential bias. The performance of the sequential and non-sequential Inbuilt Rubric method versions is very similar.

7 Maximum likelihood estimator showed appropriate model fit for CFAs of human rater scores, but the models did not converge for the Inbuilt Rubric method scores. Some differences between maximum likelihood and ordinary least squares methods have been associated with weak common factors, and the latter is recommended when substantial differences are found (e.g., Briggs & MacCallum, 2003). This lack of common factors for the computational scores could be attributed to the Gram–Schmidt algorithm used to generate Inbuilt Rubric scores.

8 Additionally, we computed Pearson correlation coefficients between the Inbuilt Rubric method scores generated with two random partitions of the original semantic space as a proxy of their reliability. For this purpose, we generated two linguistic corpora randomly splitting the Spanish Wikipedia corpus. We transformed each of these semantic spaces into its corresponding meaningful semantic space, namely classic/bifactor and descriptors/fragments for each of the three instructional texts. This makes a total of 24 new semantic spaces that were used to compute the reliability estimates as Pearson correlation coefficients between the Inbuilt Rubric method scores. We evaluated the summaries using these new meaningful semantic spaces and then we correlated the scores of both random semantic spaces. Pearson correlation coefficients showed reliable Inbuilt Rubric method scores. These results can be found in the OSF project of this paper.

9 Additional analyses were conducted to determine whether the general factor of the bifactor Inbuilt Rubric method had a transverse relation with all the specific human rater factors. In these analyses, factor loadings between the general factor of the Inbuilt Rubric method and both the general and specific human rater factors were specified. These models did not converge. This could mean that the common variance is well distilled in both the Inbuilt Rubric and the human rater general factors, and that the specific factors have remarkable topic-identity.
